# Verrucous Carcinoma of the Foot with Bone Invasion: A Case Report

**DOI:** 10.1155/2013/135307

**Published:** 2013-04-09

**Authors:** C. Pempinello, A. Bova, R. Pempinello, R. Luise, G. Iannaci

**Affiliations:** ^1^Department of Orthopaedic and Traumatology, S. Gennaro Hospital, Via San Gennaro dei Poveri, 25-80125 Napoli, Italy; ^2^Department of Infectious Diseases, Cotugno Hospital, Via G. Quagliariello, 54-80131 Napoli, Italy; ^3^Department of Pathology, Incurabili Hospital, Via Maria Longo, 50-80138 Napoli, Italy

## Abstract

Verrucous carcinoma of the foot often affects deep structures such as tendons, muscles, or bones. A 74-year-old man presented with a foot lesion that had been diagnosed as a skin infection 7 years earlier. He was treated with multiple excisions and superficial biopsies associated with antibiotic therapy without success. In our department he underwent an aggressive and accurate debridement with marginal excision harvesting multiple biopsies. Pathological evaluation of tissue at the time of operation confirmed the diagnosis of verrucous carcinoma of the foot. Therefore, the patient underwent an amputation below knee, and there were no postoperative complications; the patient was able to walk with the aid of a prosthesis with no signs of recurrence. The lesion follows a chronic course evolving from a discrete focal lesion to a large fungating deeply penetrating mass often compromised by local infection. The slow growth and confusing early-stage appearances can lead to delays in diagnosis of 8 to 15 years causing the extracutaneous involvement that requires a leg amputation. Many patients are initially treated with many topical medications without success, and most tumors have been treated as recalcitrant warts or corns for some time, whereas the basic approach is surgical.

## 1. Introduction

Verrucous carcinoma is a rare, locally invasive, well-differentiated, and low-grade squamous cell carcinoma, with low metastatic potential. It has a variety of different names; each is distinguished by its different location but represents the same pathological condition. These terms include epithelioma cuniculatum plantare, giant condylomata accuminata of the anorectal region (Buschke-Lowenstein tumour), verrucous carcinoma of the oropharynx, papilloma cutis carcinoids, epithelioid tumour, and cutaneous squamous carcinoma [1].

## 2. Case Report

**Figure 1:**
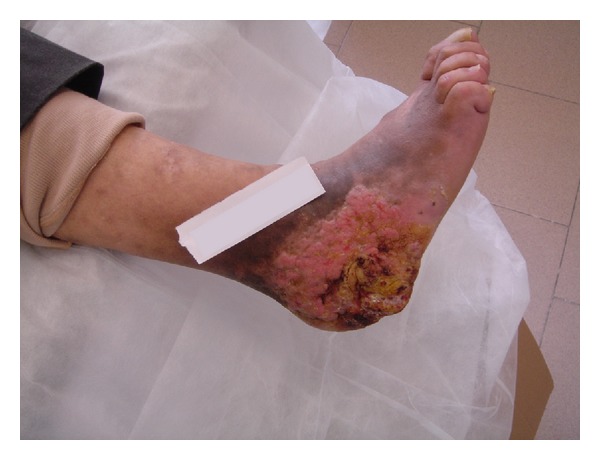
Large ulcerating lesion on the plantar surface of the calcaneus of the right foot.

**Figure 2:**
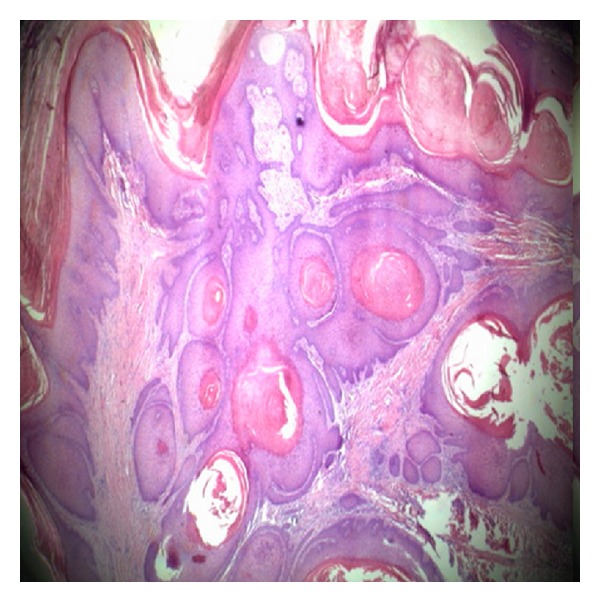
Verrucous carcinoma. This biopsy specimen shows well-differentiated neoplastic nests of squamous epithelium extending into the dermis.

**Figure 3:**
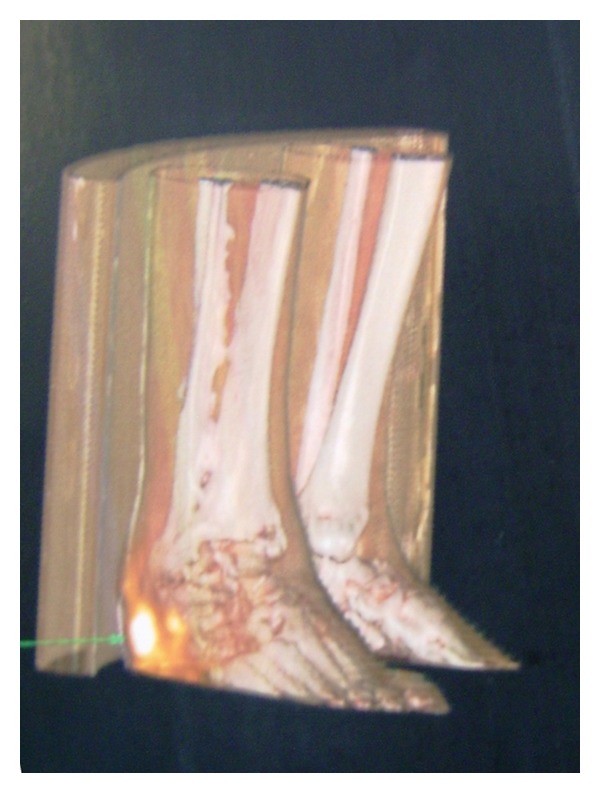
CT scan 3D of lower limb.

We report the case of a 74-year-old farmer, with a 6-year history of a large ulcerating lesion on the plantar surface of the calcaneus of his right foot (Figure 1). In 1978, thirty years earlier, he fell from a tree reporting a multiple vertebral fractures with spinal lesions. He underwent a surgical operation with reduction and fixation of the vertebral fractures with plate and screws. Later on, the vertebral fractures healed, but he recovered with a permanent urinary catheter for incontinence caused by spinal lesion. During May 2003 the patient presented with a superficial wound 1 cm large on the plantar surface of the calcaneus. Therefore, he underwent several local treatments at other institutions including multiple excisions, superficial biopsies, and targeted parenteral antibiotic therapy. The cultural specimen showed growth of *Proteus mirabilis,* and the histological examination showed only epithelial hyperplasia with no evident signs of malignancy. However, topic treatment and antibiotic treatment produced only limited improvements and the lesion was progressively enlarging and worsening. In January 2011 he was admitted to our department; the physical examination showed a worsening ulcerated verrucous plaque on the calcaneus of the right foot. No other verrucous lesions were noted on the foot. Previous treatments include multiple excisions, antibiotic therapy, and topical agents for verruca vulgaris, which produced only limited improvement. He underwent an aggressive and accurate debridement with marginal excision harvesting multiple biopsies. Pathological evaluation of tissue at the time of operation confirmed the diagnosis of verrucous carcinoma of the foot. Morphologic picture showed a large deep biopsy occupied by an ulcerated, polypoid mass characterized superficially by hyperkeratosis, parakeratosis, and acanthosis; deeply the tumor invaded with broad strands that often contained keratin-filled cysts in their center (Figure 2). Mitotic activity was low and confined in basal layer. The fibrous stroma surrounding the lesion revealed ectatic vessels, moderate inflammatory infiltrate with neutrophils and focal necrosis. The definitive histopathological diagnosis was verrucous carcinoma, that is, low-grade squamous cell carcinoma. The tumor infiltrated the soft periosteal tissues. High-quality radiographs and CT scan provided additional information regarding the involvement of the calcaneus. The tumour had expanded beneath the bone as confirmed by CT scan (Figure 3). Therefore, the patient underwent an amputation below knee and there were no post-operative complications. Later on, the patient was able to have a full extension and flexion of the knee. Three years later, the patient was walking with a leg prosthesis without any relapse of infection or recurrence.

## 3. Discussion

Verrucous carcinoma of the foot is an uncommon low-grade variant of squamous cell carcinoma characterized by local aggressive clinical behavior but a low potential for metastasis. It occurs mainly in men. The patients are predominantly older, with a mean age of 52–60 years. It is most often seen on the sole [2]. The ball of the foot is the most frequent location [3]. A high index of suspicion is necessary for the diagnosis of verrucous carcinoma related to a skin infection [4], so that it is essential that a careful history has to be obtained to assess host risk factors and wound healing [5]. The lesion follows a chronic course evolving from a discrete focal lesion to a large fungating deeply penetrating mass, often complicated by local infection [6, 7]. Because the foot has thin skin, subcutaneous tissue, and small muscles, palpation of the tumor is relatively easy [8]. Differential diagnoses include common benign lesions, actinomycosis, verruca plantaris, pseudoepitheliomatous hyperplasia, and plantar fibromatosis [9]. Malignant lesions are especially uncommon in the foot and ankle, but they often are confused with common benign lesions and, therefore, misdiagnosed (e.g., plantar sarcomas as fibromatosis, dorsal synovial sarcomas as ganglions, malignant melanomas as chronic ulceration, and primary bone tumors as stress fractures) [10]. Actinomycosis, a chronic bacterial infection caused by Gram-positive Bacilli (*Actinomyces *sp.), can present with fistulous tract lesions. Sulfur granules similar to the keratogenous material in the verrucous carcinoma may be present. Verruca plantaris is common solitary lesion on the sole of the foot. The overlying skin is typically a thickened cornified layer. Pseudoepitheliomatous hyperplasia is a pathologic reaction pattern of squamous epithelium that usually occurs in association with certain neoplasms or over a chronic inflammatory process (i.e., following trauma or irritation). The reactive epithelium may extend into the superficial reticular dermis, simulating a carcinoma. Plantar fibromatosis is a rare, benign neoplasm of the plantar aponeurosis. It is a well-circumscribed, indurated mass usually detectable with palpation. It only invades locally but has a tendency to recur after adequate surgical or corticosteroid therapy [11]. Verrucous carcinoma grows slowly and rarely metastasizes but displays locally aggressive features. It is found most commonly on the sole or ball of the foot also on knee, leg and web spaces of the toes [12, 13]. Foul-smelling keratogenous material may be excreted through multiple sinus openings. Most tumors have been treated as recalcitrant warts or corns for some time. Excision is the treatment of choice because of local aggressiveness and infrequent metastasis. Verrucous carcinoma is a low-grade, locally invasive tumour, which almost never metastasizes and thus has a favourable prognosis. The recommended treatment is wide local excision, rather than marginal excision, as verrucous carcinoma often causes a structural distortion of adjacent tissues, and the margins are not always apparent intraoperatively. The residual defect can then be covered with a full thickness skin graft or radial forearm free flap. Other therapeutic modalities include topical chemotherapy, electrocautery, cryotherapy, and laser therapy, but all have high recurrence rates, whereas radiotherapy is not recommended because of the possibility of malignant changes. Computed tomography is superior to MRI in determining minimum changes in the cortical bone related to tumor invasion. CT scan is a good alternative to determine incipient bone invasion [14]. Early diagnosis of verrucous carcinoma in our patient has lead to a more favourable treatment, but late diagnosis has caused deep bone invasion that unfortunately requested in some cases of leg amputation below knee [15]. The slow growth and confusing early-stage appearances can lead to delays in diagnosis of several years and hence undertreatment.
